# The importance of environmental conditions in maintaining lineage identity in *Epithelantha* (Cactaceae)

**DOI:** 10.1002/ece3.7347

**Published:** 2021-03-11

**Authors:** David Aquino, Alejandra Moreno‐Letelier, Miguel A. González‐Botello, Salvador Arias

**Affiliations:** ^1^ Jardín Botánico Instituto de Biología Universidad Nacional Autónoma de México Ciudad de México México; ^2^ Sociedad de Cactáceas y Suculentas del Estado de Nuevo León Guadalupe México

**Keywords:** ancestral state reconstruction, bioclimatic variables, Cactaceae, *Epithelantha*, soil variables, species delimitation

## Abstract

- The use of environmental variables to explain the evolution of lineages has gained relevance in recent studies. Additionally, it has allowed the recognition of species by adding more characters to morphological and molecular information. This study focuses on identifying environmental and landscape variables that have acted as barriers that could have influenced the evolution of *Epithelantha* species and its close genera.
- Our results show that soil pH, isothermality, temperature seasonality, and annual precipitation have a significant phylogenetic signal for *Epithelantha*. Soil type and landforms are also relevant as ecological barriers that maintain the identity of *Epithelantha* species.
- The variables associated with the soil (pH) have influenced the evolution of *Epithelantha* and probably in other genera of Cactaceae. Additionally, *Epithelantha* is frequent in the piedmont and haplic kastanozems. Bioclimatic variables reinforce the recognition of *E. micromeris,* and *E. cryptica* as independent species. Therefore, ecology can be considered as a factor to explain the high level of endemism in Cactaceae.

## Graphical abstract

Speciation and species delimitation in megadiverse groups has always been a challenge. Here, we analyzed the impact of environmental conditions in maintaining lineage identity in an endemic cactus.

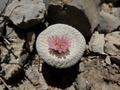

## 1 INTRODUCTION

The study of diversification processes in megadiverse groups has been strengthened by the inclusion of bioclimatic and other environmental information (Jones et al., 2014; Nevado et al., 2018). The Cacteae tribe has nearly 384 species and a high number of endemisms (Vázquez‐Sánchez et al., 2013). This tribe diversified due to climate changes; however, there are no studies focusing on which specific biotic or abiotic factors contributed to its diversification and infer the speciation process (Hernández‐Hernández et al., 2014). Previously, the study of environmental tolerances of members of Cactaceae has been applied in conservation studies of *Disocactus phyllanthoides* (Iberri, 2009), *Echinocereus reichenbachii* (Butler et al., 2012), *Astrophytum coahuilense* (Cardoza‐Martínez et al., 2019), and recently, in the influence of temperature in limiting the distribution of *Thelocactus* and the *Stenocereus griseus* species complex (Alvarado‐Sizzo et al., 2018; Mosco, 2019). Environmental information has also been used to understand the divergence and speciation in genera such as *Eriosyce* subgenus *Neoporteria* (Guerrero et al., 2011, tribe Notocacteae) and in *Rapicactus* and *Turbinicarpus* (Cacteae), which had been previously treated as synonyms (Donati et al., 2017).

Soil characteristics are factors that could also influence lineage diversification in plants (Anacker & Strauss, 2014). Soil plays an essential role in determining the distribution of plant communities and in consequence the distribution of endemic taxa (Bárcenas‐Argüello et al., 2013; Burge, 2014). Del Castillo (1996) identified species as calcareous‐tolerant and calcareous‐intolerant, being calcareous soils the ones that harbor more endemism. Soil characteristics have been identified as a limiting factor in the distribution of *Echinocactus platyacanthus* (Trujillo, 1984), and *Ariocarpus* (Aguilar‐Morales et al., 2011). This condition is not exclusive to Cacteae, as it has been reported in *Cephalocereus* (Tribe Echinocereeae, Bárcenas‐Argüello et al., 2010), and *Opuntia* plus *Cylindropuntia* from the Chihuahuan desert (Subfamily Opuntioideae; Lebgue‐Keleng et al., 2014).

**FIGURE 1:**
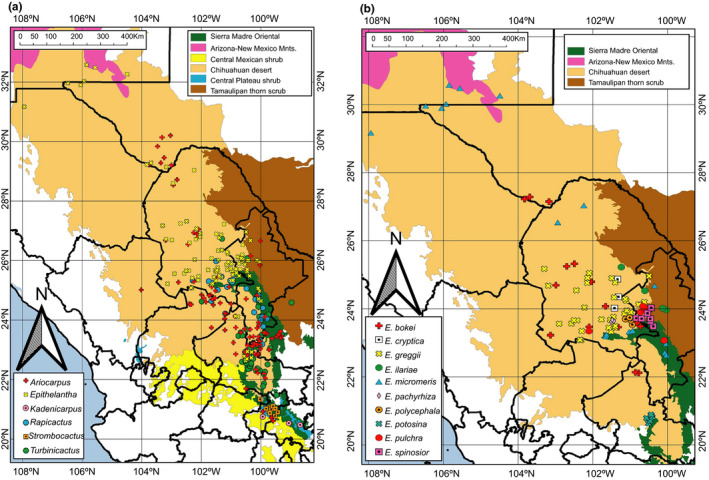
(a) Distribution of *Epithelantha* and closely related genera, based on georeferenced records for this work. (b) Distribution of species within *Epithelantha*

To study the impact of environmental factors on speciation, we focused on the genus *Epithelantha,* which due to its moderate diversity and endemicity can serve as a model without the confounding factor of geographic distance. This genus is nested in a clade composed of *Ariocarpus*, *Rapicactus*, *Strombocactus*, *Turbinicarpus*, and *Kadenicarpus* (Aquino et al., 2019; Vázquez‐Sánchez et al., 2019). The six genera are mainly distributed in the ecoregion called the Chihuahuan desert, and neighboring regions in North and Northeastern Mexico (Figure 1a). *Epithelantha* is the subject of several taxonomic controversies. Aquino et al. (2019) recognized 10 species based on a combination of morphological and molecular characters (Figure 1b). However, *E. micromeris* and *E. cryptica*, can only be identified by morphological characters, thus are good candidates to test the relevance of environmental factors maintaining species identity (Rissler & Apodaca, 2007). Environmental information has been used to identify cryptic species of other plant groups such as *Leucanea* (Fabaceae, Govindarajulu et al., 2011) and *Nolina* (Asparagaceae, Ruíz‐Sánchez & Specht, 2014). More recently, Alvarado‐Sizzo et al. (2018) used information of species distribution and climate to formulate hypotheses of reproductive isolation in *Stenocereus griseus* complex.

This study aims at understanding the role of the environment at promoting species boundaries in the genus *Epithelantha*. Out main objectives are: (a) to characterize climate and soil niche variables for *Epithelantha* and other closely related genera by extracting data from geographic information systems; (b) to define whether the distribution of *Epithelantha* species is associated to physical‐ecological barriers that maintain the identity of lineages by contingency tests and ancestral state reconstruction; and (c) to corroborate the presence of environmental differences between *E. micromeris* and *E. cryptica*.

## 2 MATERIALS AND METHODS

### 2.1 Taxonomic considerations

The species of *Epithelantha* used in this study are: *E. bokei* L. D. Benson*, E. cryptica* D. Donati & Zanov.*, E. greggii* (Engelm.) Orcutt*, E. ilariae* D. Donati & Zanov.*, E. micromeris* (Engelm.) Britton & Rose*, E. pachyrhiza* (W.T. Marshall) Backeb.*, E. polycephala* Backeb., *E. potosina* (D. Donati & Zanov.) D. Aquino & S. Arias*, E. pulchra* (D. Donati & Zanov.) D. Aquino & S. Arias, and *E. spinosior* C. Schmoll (Aquino et al., 2019). For the *Ariocarpus*, *Kadenicarpus*, *Lophophora, Echinocactus*, *Rapicactus*, *Strombocactus*, and *Turbinicarpus* circumscription we follow Donati et al. (2017) and Vázquez‐Sánchez et al. (2019, 2013), *Coryphantha* follows Vázquez‐Benítez et al. (2016), and for *Mammillaria,* we followed the compilation of Hunt et al. (2006).

### 2.2 Raw geographic and environmental data

In order to characterize the environmental preferences each taxon, presence records for *Epithelantha* F.A.C. Weber ex Britton & Rose, *Ariocarpus* Scheidw.*, Kadenicarpus* Doweld*, Rapicactus* Buxb. & Oehme*, Strombocactus* Britton & Rose, and *Turbinicarpus* (Backeb.) Buxb. & Backeb., were obtained from 15 herbaria (ANSM, ASU, CFNL, DES, ENCB, GBH, IEB, NCU, MEXU, MO, QMEX, SLPM, UAMIZ, UNL, and US, following the nomenclature in Thiers, 2015). Except for *Kadenicarpus* and *Strombocactus*, field work was carried out to complement the geographical information of the remaining four genera in the states of Coahuila, Nuevo León, San Luis Potosí, and Tamaulipas. Duplicate and imprecise localities were removed from the database, as well as those found less than 5 km from each other. Geographic distances were estimated with Geographic Distance Matrix Generator 1.2.3 (Ersts, 2018). The final database included 101 records for *Epithelantha,* 91 for *Ariocarpus,* 26 for *Rapicactus,* 11 for *Strombocactus*, 60 for *Turbinicarpus* and 6 for *Kadenicarpus*. Records corresponding to the chosen outgroup were also included: *Coryphantha* (Engelm.) Lem. (575 records), *Echinocactus* Link & Otto (111 records), *Lophophora* J. M. Coult. (71 records), and *Mammillaria* Haw. (1726 records).

Climatic characterization was done using 19 bioclimatic variables (WorldClim Global Climate 1.4; Hijmans et al., 2005; Table S2). In addition to climate, we included soil variables like soil pH, soil type, and landform (Cruz‐Cárdenas et al., 2014; Fischer et al., 2008; Pineda et al., 2014), as these have shown to be important determinants in the distribution of other plants (Anacker & Strauss, 2014). Variables were extracted using QGIS 2.1.8 (QGIS Development Team, 2009). The layers are of a spatial resolution of approximately 1 km^2^. Correlated variables were identified using the Pearson correlation coefficient (PCC; Restrepo & González, 2007), and only retaining variables with a correlation coefficient <=0.7: Bio1, Bio3, Bio4, Bio9, Bio11, and Bio12 (Dormann et al., 2013).

### 2.3 Phylogenetic reconstruction and dating

We constructed a sequence data matrix using the following cpDNA regions: *petL‐psbE, psbA‐trnH, trnL‐F,* and *trnQ‐rps16*. The DNA extraction, amplification, and sequencing methods are described in Aquino et al. (2019), and GenBank accession numbers can be found in Table S1. The coding of indels and morphological characters can be found in Aquino et al. (2019). A calibrated Bayesian phylogenetic reconstruction was constructed using a representative of each species of *Epithelantha*, plus its closest relatives: *Turbinicarpus alonsoi* Glass & S. Arias*, Kadenicarpus pseudomacrochele* (Backeb.) Doweld*, K. horripilus* (Lem.) Vázquez‐Sánchez, *Rapicactus beguinii* (N.P. Taylor) Lüthy*, Ariocarpus trigonus* (F.A.C. Weber) K. Schum, and *Strombocactus disciformis* (DC.) Britton & Rose. Outgroups were: *Lophophora williamsii* (Lem. ex Salm‐Dyck) J.M. Coult.*, Coryphantha calipensis* Bravo ex S. Arias, U. Guzmán & S. Gama‐López*, Mammillaria mystax* Mart., and *Echinocactus platyacanthus* Link & Otto. The alignment included a total of 20 taxa and 3,736 sites, a total of 1,061 sites without gaps or missing data and 185 polymorphic sites. Two calibration points were used following Hernández‐Hernández et al. (2014). One placing the divergence between *Echinocactus* and Mammilloid clade at 15.27 Mya (HDP95%‐10.94–21.85), and a second was the divergence between Mammilloid clade (*Coryphantha* and *Mammillaria*) and *Lophophora*, with *Epithelantha, Turbinicarpus, Kadenicarpus, Rapicactus, Ariocarpus,* and *Strombocactus* (8.62 Mya; HDP 95%‐ 5.83–12.56). A normal distribution was used in most cases, with a mean set at the calibration point and a standard deviation of 2. The evolution model was GTR + G, and lognormal molecular clock model and Yule speciation model were used. The analysis was performed using BEAST 1.7.4 (Drummond et al., 2012), with 10 million chains and 10% burnin, and results were analyzed with Tracer 1.7 and TreeAnnotator 1.7 once all ESS values were above 300 (Drummond et al., 2012; Rambaut et al., 2018). The final alignment and trees are available in the Dryad repository (https://doi.org/10.5061/dryad.0gb5mkm0b).

### 2.4 Environmental characterization

Categorical variables (soil type and landform) were analyzed using a contingency matrix, registering the frequency of observations of each species for each variable. Based on that, the expected observation matrix and Pearson residuals were calculated to apply the chi‐squared goodness of fit test with the R package *dplyr* and plotted with *corrplot* (Gómez‐Gómez et al., 2003; Wei & Simko, 2017; Wickham et al., 2020). The test was performed with all the records for each genus: *Epithelantha, Ariocarpus, Kadenicarpus, Rapicactus, Strombocactus,* and *Turbinicarpus*. Another test was performed for each species within *Epithelantha*.

The bioclimatic variables that were used for ancestral state reconstruction were chosen by first evaluating their independence using Pearson's correlation coefficients (Table S2). The variables used were annual mean temperature, isothermality, temperature seasonality, mean temperature in the driest quarter, mean temperature of the coldest quarter, annual precipitation, and soil pH. In all cases, the mean was estimated and was used to reconstruct the ancestral states based on the calibrated phylogenetic tree (Table S3). The reconstruction of ancestral states was performed with the R package *phytools* (Revell, 2012; R Development Core Team, 2018). Additionally, phylogenetic signal was estimated with Blomberg's K and Pagel's Lambda (Revell, 2012).

Ancestral state reconstruction of discrete variables (landform and soil type) was performed by first evaluating character evolution models using fitpolyMK implemented by phytools (Revell, 2012). First, we reduced the number of character states by keeping only those states that were represented in 70% of the sample. For soil type we had kastanozems, lithosols and xerosols; and for landforms, the final character states were fold mountains, karstic formations, river systems, plains, and piedmonts. The data was considered unordered since at least one taxon had more than 2 character states. We used the function fitpolyMK to test for two models: ER and transient, and for each dataset we kept the model with the lowest AIC. The best fitting model for soil type was ER‐unordered and for landforms was transient‐unordered. We used the state transition matrix generated by fitpolyMK to reconstruct the ancestral character states using make.simmap for 100 trees.⁠⁠

## 3 RESULTS

### 3.1 Phylogenetic reconstruction

**FIGURE 2:**
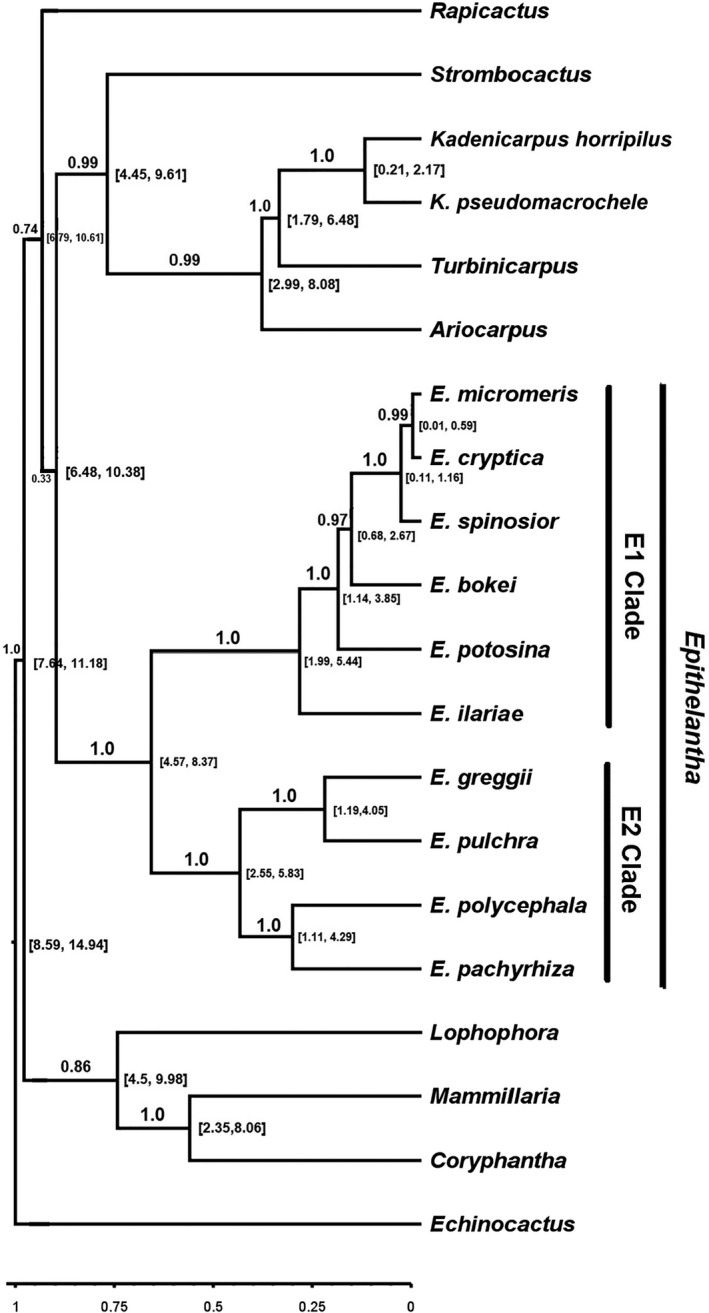
Tree calibrated for *Epithelantha* and allied genera (Cacteae, Cactaceae) applying Bayesian inference. The bold values on the branches correspond to the posterior probability values and the values in square brackets on the nodes indicate the approximate age range of divergence

The genus *Epithelantha* diverged from its sister group 9.09 Mya (6.48–10.38 Mya 95% HPD). Within *Epithelantha*, two clades diverged 6.71 Mya (4.57–8.37 Mya; 95% HPD), with the clade E2 including *E. greggii, E. pachyrhiza, E. polycephala* and *E. pulchra* diverging 4.53 Mya (2.55–5.83 Mya; 95% HPD). The other clade, E1, diverged 3.03 Mya (1.99–5.44 Mya; 95% HPD) and includes *E. bokei, E. cryptica, E. ilariae, E. micromeris, E. potosina,* and *E. spinosior* (Figure 2).

### 3.2 Environmental characterization

#### 3.2.1 Categorical variables

**FIGURE 3:**
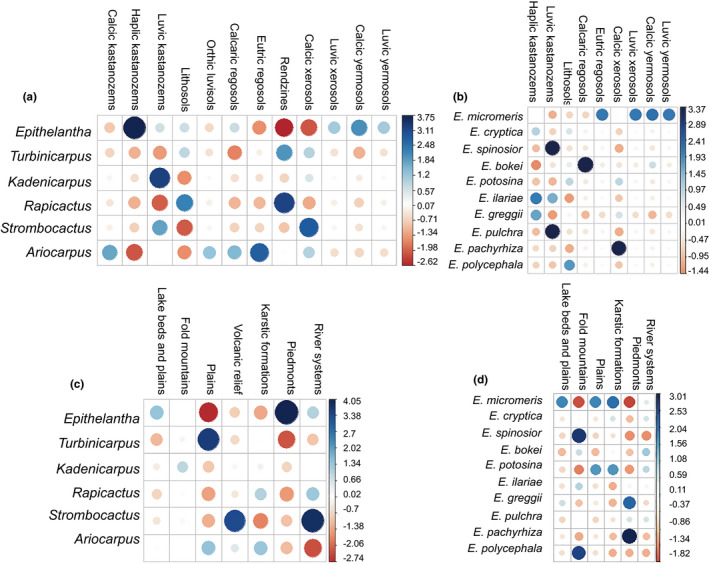
Correlograms between: (a) Soil type and genus; (b) Soil type and species of *Epithelantha*; (c) Landform and genus; (d) Landform and species of *Epithelantha*. Positive correlations are shown in blue colors and negative in red colors. Residuals are shown in the right hand side of each plot

The association between soil type and *Epithelantha's* sister genera was significant (chi‐squared *p* = 4.246e^−08^, Figure 3a). *Epithelantha* shows an association to haplic kastanozems (Pearson's *r* = 3.749) and a negative association with rendzines (Pearson's *r* = −2.617). *Turbinicarpus* has a moderate correlation to rendzines (Pearson's *r* = 1.95). For the other genera, *Kadenicarpus* is found in luvic kastanozems (Pearson's *r* = 3.224); *Rapicactus* is also frequent in rendzines (Pearson's *r* = 3.192) and lithosols (Pearson's *r* = 2.231); *Ariocarpus* is found in eutric regosols (Pearson's *r* = 2.754) and calcic kastanozems (Pearson's *r* = 1.761) and a negative association with haplic kastanozems (Pearson's *r* = −2.078). Finally, *Strombocactus* is mostly found in calcic xerosols (Pearson's *r* = 2.848) and luvic kastanozem (Pearson's *r* = 1.173), with a negative correlation with lithosol (Pearson's *r* = −2.053).

The goodness of fit test applied to evaluate the association between species and soil type was significant (*p* = .00269, Figure 3b). The highest correlation value was for *Epithelantha pachyrhiza's* (Pearson's *r* = 3.372) association with calcic xerosols. *E. bokei* is commonly found in calcaric regosols (Pearson's *r* = 3.662), while *E. pulchra* y *E. spinosior* is found in luvic kastanozems (Pearson's *r* = 3.36). *E. micromeris* is associated with calcic yermosols (Pearson's *r* = 2.212), eutric regosols, luvic xerosols and luvic yermosols (Pearson's *r* = 2.114 for each). Meanwhile, its sister species *E. cryptica* showed no significant association with any type of soil in particular. *E. ilariae* and *E. greggii* are often found in haplic kastanozems (Pearson's *r* = 2.07 y 1.647, respectively). The association of *E. polycephala* with lithosols is weak (Pearson's *r* = 1.76), and for *E. potosina* there is no significant association with any soil type.

The test between genera and landforms was also significant (*p* = 6.943^e−10^, Figure 3c). *Epithelantha* is commonly found in piedmonts (Pearson's *r* = 4.055) and has negative association with plains (Pearson's *r* = −2.737). In contrast, *Turbinicarpus* is mostly found in plains (Pearson's *r* = 3.578) with a negative association with piedmonts (Pearson's *r* = −2.3). *Strombocactus* is found in river systems (Pearson's *r* = 3.827), while *Ariocarpus*, *Kadenicarpus,* and *Rapicactus* show no significant association with a particular landform. The goodness of fit for species of *Epithelantha* was significant for landforms (*p* = .008966, Figure 3c). The species found in piedmonts were *Epithelantha pachyrhiza* (Pearson's *r* = 3.009) and *E. greggii* (Pearson's *r* = 2.121). *E. polycephala* (Pearson's *r* = 2.58), *E. spinosior* (Pearson's *r* = 2.719), and *E. ilariae* (Pearson's *r* = 0.745) are frequent in fold mountains. *E. potosina* is mostly associated with karstic formations (Pearson's *r* = 1.569) and plains (Pearson's *r* = 1.564). Meanwhile, *E. micromeris* is also frequent in karstic systems (Pearson's *r* = 2.014), lake beds/plains, and plains (Pearson's *r* = 1.716 in both cases). Additionally, there was a negative association between the later species with two landforms: piedmont (Pearson's *r* = −1.824) and fold mountains (Pearson's *r* = −1.721).

### 3.3 Phylogenetic signal and ancestral state reconstruction

**FIGURE 4:**
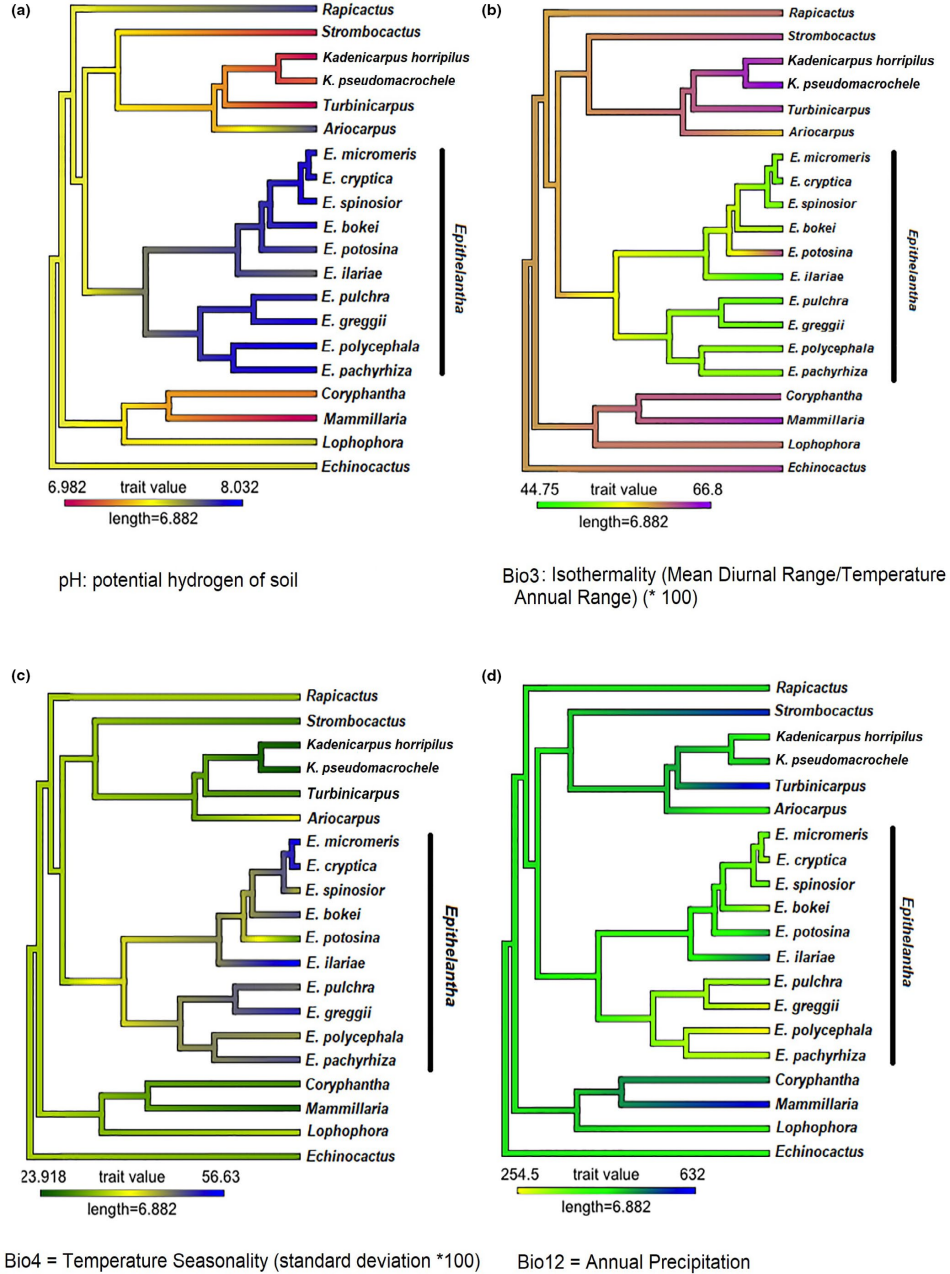
Ancestral character state reconstruction for four environmental variables whose phylogenetic signal was significant

**FIGURE 5:**
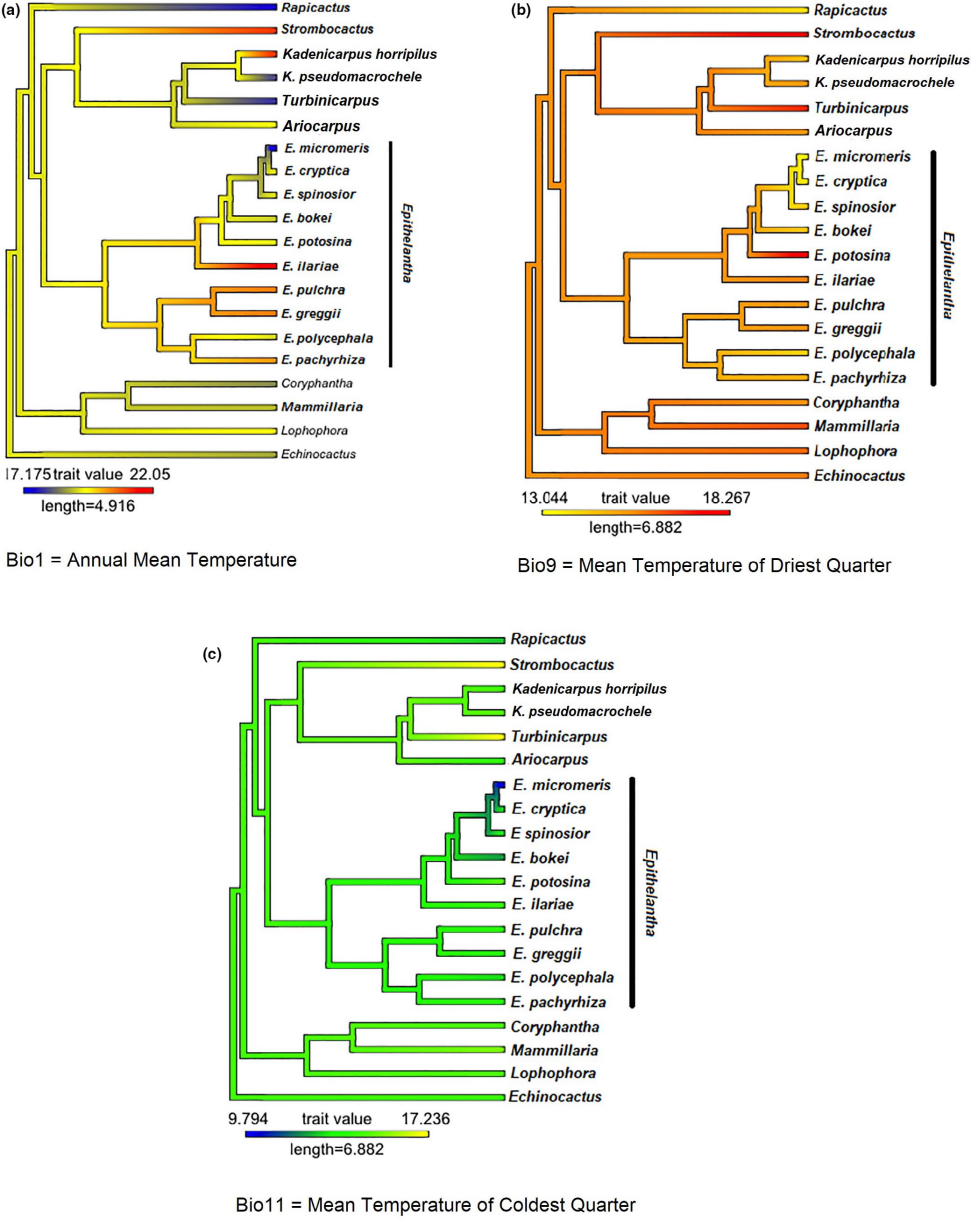
Ancestral character state reconstruction for three environmental variables whose phylogenetic signal was not significant

The estimated Blomberg's K and Pagel's *λ* were significant for Bio3 (isothermality, *K* = 1.164444; *λ* = 0.9818318), Bio4 (temperature seasonality, *K* = 0.8811825; *λ* = 0.7242332), Bio12 (annual precipitation, *K* = 0.7216596; *λ* = 0.8884284), and soil pH (*K* = 1.511694; *λ* = 1.022611). The ancestral state reconstruction of variables can be seen in Figure 4, where the genus *Epithelantha* shows higher pH values (7.48–8–03) overall. The exception is *E. ilariae* with the lowest average (pH = 7.84), similar to *Ariocarpus* (pH = 7.81) and *Rapicactus* (pH = 7.84). The genera *Kadenicarpus, Turbinicarpus,* and *Strombocactus* are found in neutral soils (pH = 7.01–7.18) (Figure 4b). Regarding isothermality (Bio3 = 49.31–51.56), *Epithelantha* has the lowest values, which means that temperature is more variable (Figure 4c), with the exception of *E. potosina*, which has an isothermality similar to the sister genera (*Ariocarpus, Kadenicarpus, Strombocactus* and *Turbinicarpus*). The same pattern can be seen for temperature seasonality (Bio4), with *E. potosina* again having the lowest values similar to *Turbinicarpus* (Figure 4c). Annual precipitation shows that the ancestral state for *Epithelantha* is 311–482 mm, however, the clade that includes *E. greggii* and *E. polycephala* shifts to lower precipitation levels (245–302 mm), while *E. ilariae* (Bio12 = 544 mm) prefers sites with higher precipitation (Figure 4d), like *Strombocactus* (Bio12 = 586 mm) and *Turbinicarpus* (Bio12 = 632 mm). Nonsignificant phylogenetic signal was found for Bio1 (Annual mean temperature, *K* = 0.1694556; *λ* = 6.761339e^−05^), Bio9 (Mean temperature of driest quarter, *K* = 0.4216433; *λ* = 6.761339e^−05^) and Bio11 (Mean temperature of coldest quarter, *K* = 0.264773; *λ* = 0.4094559). Reconstruction of ancestral character states can be seen in Figure 5a–c. In this case, two sister species (*E. cryptica* and *E. micromeris*) show a marked divergence in character states for Bio1 and Bio11 (Figure 5a,c).

**FIGURE 6:**
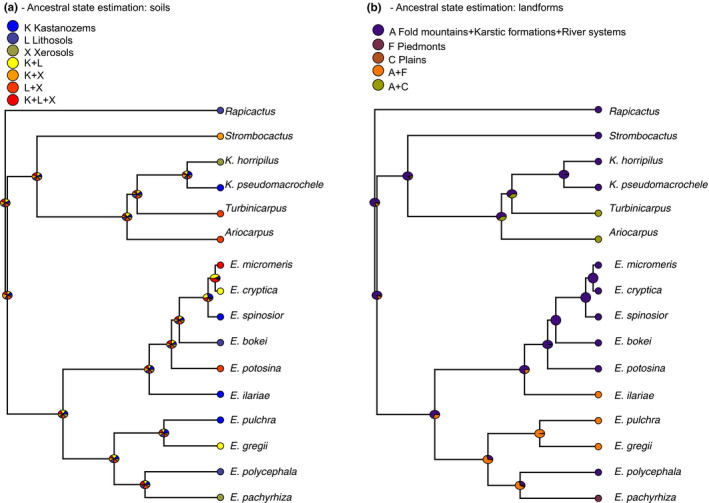
Ancestral character state reconstruction of soil types and landforms for *Epithelantha* and sister genera

The ancestral state reconstruction model fitting for soil type showed a complex pattern due to the high levels of intraspecific polymorphism and the unordered nature of characters (Figure 6a, Figure S1a,b). Soil type in particular showed no phylogenetic signal, as all soil types were present in the genus *Epithelantha* and the outgroup. However, there were differences between sister species, like *E. polycephala* (lithosols) and *E. pachyriza* (xerosols) (Figure 6a). The results for landforms were even more complex, due to the high number of character states (Figure 6b, Figure S1b). For ease of representation, we grouped the three more widely represented landforms (fold mountains, karstic formations, and river systems) into one single category used only for plotting purposes. That highlights the presence of piedmonts exclusively in *Epithelantha*, and plains in the outgroup (Figure 6b). Just as we observed for soil types, the sister species *E. polycephala* and *E. pachyriza* are found in different landforms (fold mountains and piedmonts respectively) and are not polymorphic (Figure 6, Figure S1c).

## 4 DISCUSSION

Our results show that *Epithelantha* has a preference for calcareous soils, with higher pH and drier more seasonal climate than its closely related genera. Within the genus, several species showed a preference for different environmental conditions related to temperature for sister species *E. micromeris*, *E. cryptica*, and soil type and landforms for *E. polycephala* and *E. pachyrhiza*. These environmental differences between sister species suggest differential selection pressures and local adaptation, which could have driven the speciation process (Levin, 2004; McCormack et al., 2009).

The results of our phylogenetic analyses showed that *Epithelantha* diverged ca. 9 Mya in the Miocene, which coincides with the estimated date of diversification of Cactaceae, and the aridification and establishment of the vegetation of the Chihuahuan desert (from mid‐Miocene to late Pliocene; Arakaki et al., 2011; Hernández‐Hernández et al., 2014; Scheinvar et al., 2020; Van Devender, 1990). During this time, the deserts of North America developed and there was an increase in species richness in the region (Wiens et al., 2013). Most of the species within the genus are younger than 2.5 My, which coincides with the onset of the climatic oscillations of the Pleistocene which had a strong effect on the Chihuahuan Desert biota (Ezcurra et al., 2020; Gámez et al., 2017; Sánchez‐Escalante et al., 2005; Scheinvar et al., 2020).

### 4.1 Influence of soil and bioclimatic variables on the evolution of *Epithelantha*

Soil conditions are among the factors that determine the distribution of species in Cactaceae. Similar patterns have been observed in other groups, for example, the ability to grow in serpentine soils is considered to be plesiomorphic for *Oxera* (Lamiaceae) and *Guioa* (Sapindaceae; De Kok, 2002), and an affinity for gypsum has promoted the differentiation and high levels of endemicity of desert flora in the Cuatro Ciénegas basin (Aguirre‐Liguori et al., 2014; Ochoterena et al., 2020). The current distribution of *Epithelantha* is limited to the Eastern Chihuahuan desert (Figure 1), and this distribution is the consequence of the interaction of soil and climate with historical factors. Our results show that soil characteristics, and not climate, are the most important factors limiting the distribution of *Epithelantha* compared with closely related genera (Figure 3a–c). The most common soil type where *Epithelantha* occurs is haplic kastanozems (Figure 3b), which are characterized by accumulation of carbonates and a reduced layer of humus, as well as in piedmonts (Fischer et al., 2008). The pH conditions where species are commonly found go from 7.84 to 8.04, in contrast with other genera which grow in more acidic soils (Figure 4a). These later patterns suggest that the colonization of alkaline soils could have driven the differentiation of *Epithelantha* from its sister genera. Four species show a strong association with haplic kastanozems: *E. bokei, E. pachyrhiza, E. pulchra* and *E. spinosior* (Figure 3b). Only *E. bokei* is broadly distributed in the Chihuahuan desert, while *E. pulchra* and *E. spinosior* are found at the foothills of pine‐oak forests in the Sierra Madre Oriental, and *E. pachyrhiza* has the smallest distribution range of the genus (Figures 1, 3). Close association with a specific soil type has been reported for *Cephalocereus totolapensis,* found in a pH range of 5.5–6–9 (Bárcenas‐Argüello et al., 2010), while *C. parvispinus* is found in gypsum‐rich soils (Arias et al., 2019), where the pH is more alkaline, thus suggesting that the soil type may have a similar role in the diversification of *Epithelantha* (Figure 4a). A consequence of the interaction soil–plant is the incorporation of large quantities of biominerals, mainly calcium oxalates, which accumulate in various tissues of *Turbinicarpus* and *Rapicactus,* and can be phylogenetically informative, as each lineage has different occurrence and structure (De la Rosa‐Tilapa et al., 2018). Therefore, it is possible that *Epithelantha* has similar structures, as it is found in calcium carbonate‐rich soils.

The use of phylogenetic comparative methods broadens our understanding on how plant lineages evolved under changing selection pressures (Bárcenas‐Argüello et al., 2013). The distribution of species in the Chihuahuan desert has been deeply influenced by climate changes starting in the Miocene and throughout the Pleistocene (Scheinvar et al., 2016). Therefore, it follows that the main bioclimatic variable that determines the distribution of *Epithelantha* is isothermality (Bio3, mean 51.23), with lower values than its sister genera (Figure 4b). Within the genus, *E. potosina* has the highest value, which is due to this species being found in tropical latitudes, while the rest of the species are distributed north of the Tropic of Cancer, thus experiencing higher seasonal variations (Figure 1b). Likewise, the isothermality value of *E. potosina* is similar to those observed for *Turbinicarpus* (Figure 4b), also found in lower latitudes (Grupo San Luis, 2004). Another genus that shares a similar pattern with *Epithelantha* is *Thelocactus* Britton & Rose. Similar isothermal values imply that the habitat is subject to extreme temperature fluctuations (Mosco, 2019). Isothermality has also favored adaptive radiation events in *Mammillaria* (López, 2017), and together with precipitation seasonality, these variables have been involved in the differentiation of *Stenocereus* species (Alvarado‐Sizzo et al., 2018). Further exploration into other genera of Cactaceae could confirm this pattern. Annual precipitation (Bio12, O'Donnell and Ignizio, 2012) also shows significant phylogenetic signal, as well as isothermality, with only *E. greggii* and *E. polycephala* being present in areas with slightly lower precipitation values than the rest of the members of the genus (Bio12 = 254–302 mm; Figure 4d). These two species had been previously considered as conspecific, however, *E. greggii* has a broad East‐West distribution, while *E. polycephala* is restricted to the East of the Chihuahuan desert (Figure 1b). However, each species is associated to different soil types, and together with morphological and molecular information, there is enough evidence to recognize two species (Aquino et al., 2019). The other species that deviates from the mean precipitation observed in the genus is *E. ilariae* (Figure 4d), which is found in areas with higher precipitation (544 mm), in the transition between Chihuahuan desert and the Tamaulipan thorn scrub (Figure 1b).

### 4.2 Environmental variables to support species delimitation

While there were several bioclimatic variables that showed phylogenetic signal and had similar values on members of *Epithelantha*, the variables without phylogenetic signal can be more informative to detect ecological divergence between sister species. Mean annual temperature (Bio1) and mean temperature of the coldest quarter (Bio11) show this pattern (Figure 5) Both variables show climatic divergence of sister species *E. micromeris* and *E. cryptica* (Figure 5a,c). Aquino et al. (2019) showed that it is not possible to distinguish both taxa using chloroplast DNA sequences alone, requiring a combination of morphological characters to recognize them. The ancestral state reconstruction shows a divergence in climatic variables in both lineages, with *E. micromeris* preferring colder and drier places than *E. cryptica*. The tolerance for harsher environments seen in *E. micromeris* might explain its broader distribution (Wiens et al., 2013), going from the Chihuahuan desert to Sierra Madre Oriental pine‐oak forests. Additionally, *E. micromeris* is the only species found in three different landforms and four soil types, while *E. cryptica* is geographically restricted and only found in one soil type (Figure 1b).

In relation to soil properties, *Epithelantha* is most commonly found in piedmont landforms, while *Turbinicarpus* is found in plains. *E. pachyrhiza, E. polycephala,* and *E. spinosior* are more strongly associated with a single landform, with *E. micromeris, E. greggii,* and *E. potosina* following in association with specific landforms. The sister species *E. polycephala* and *E. pachyrhiza* are associated to different landform/soil combinations. *E. polycephala* is found mostly in fold mountains/lithosols and *E. pachyrhiza* is found in piedmonts/xerosols, thus highlighting the importance of differential habitat use in promoting or maintaining lineage identity (Figure 6, Figure S1). The reports of associations between cacti and landforms are scarce. Trujillo (1984) reports that the density of individuals of *Echinocactus platyacanthus* diminishes in piedmonts or plains, where the shrub component is more abundant. Meanwhile, Bárcenas‐Argüello et al. (2010) report that *Cephalocereus* is restricted to well‐drained landforms. This association between lineages and landforms/soil types is relevant because environmental variations within each edaphically diverse environment could promote isolation (Cavender‐Bares & Pahlich, 2009; Kozak & Wiens, 2006; Mendoza, 2017; Ochoterena et al., 2020). Additionally, the development of a landform implies dynamic processes like tectonism, weathering, and erosion, all affecting the structure of plant communities (Huggett, 2011). These landform dynamics have been important in the evolution of the Chihuahuan desert's flora, as small changes in moisture levels in a landform can determine the presence or absence of specific species (Wondzell et al., 1996). These shifts in distribution caused by small changes in environmental conditions could help explain the diversity and co‐occurrence of species within *Epithelantha*.

The combination of three sources of information (morphology, DNA, and environmental data) has a broad potential for species delimitation. This study confirms our hypothesis that *Epithelantha* is an environmentally divergent genus and that small but significant changes in soil conditions have influenced the species diversification (Donoghue & Edwards, 2014). Finally, the information derived from the understanding of ecological niche is important to inform conservation efforts. An important number of the species within *Epithelantha* and related genera have populations restricted to <10 km^2^ (Hernández et al., 2010). From this study, *E. pachyrhiza* is the species with the smallest distribution and more environmental restrictions, which should be considered to protect this species natural habitat. Therefore, the next step is to construct the potential distribution of species of *Epithelantha*, as well as understanding biotic interactions (pollinators, seed dispersers, and pathogens), which could justify its inclusion in the Norma Oficial Mexicana (NOM‐059‐SEMARNAT‐2010) and IUCN Red List.

## CONFLICT OF INTEREST

The authors have declared that no competing interests exist.

## AUTHOR CONTRIBUTION

**David Aquino:** Conceptualization (lead); Data curation (lead); Formal analysis (lead); Investigation (equal); Methodology (supporting); Supervision (lead); Writing‐original draft (equal); Writing‐review & editing (equal). **Alejandra Moreno‐Letelier:** Conceptualization (lead); Formal analysis (equal); Methodology (equal); Writing‐original draft (equal); Writing‐review & editing (equal). **Miguel A. González‐Botello:** Data curation (equal); Formal analysis (supporting); Investigation (supporting); Resources (supporting). **Salvador Arias:** Formal analysis (equal); Funding acquisition (lead); Project administration (lead); Resources (lead); Supervision (equal); Validation (equal); Writing‐original draft (equal); Writing‐review & editing (equal).

## Supporting information

Supplementary Material

## Data Availability

GenBank accessions MK283983–MK284218; geographical data can be consulted in the collections of herbaria listed in the methods. DNA alignment and trees can be accessed in the Dryad Repository https://doi.org/10.5061/dryad.0gb5mkm0b.
