# Supplementary material for: The importance of environmental conditions in maintaining lineage identity in Epithelantha (Cactaceae)
Source: Ecol Evol. 2021 Mar 11;11(9):4520–31. doi: 10.1002/ece3.7347 (PMC8093668; doi:10.1002/ece3.7347)
Supplement: Supplementary file 1 — Supplementary Material [file ECE3-11-4520-s001.docx]

**Supporting Information**

Article title: **The importance of environmental conditions in maintaining lineage identity in *Epithelantha* (Cactaceae)**

Authors: David Aquino, Alejandra Moreno-Letelier, Miguel A. González-Botello, and Salvador Arias.

**The following Supporting Information is available for this article**:

**Table S1** Representative specimens of *Epithelantha* species and allied examined and corresponding GenBank accession numbers (*petL-psbE, psbA-trnH, trnL-F, trnQ-rps16*). *— missing sequence. The voucher was deposited in MEXU. *Epithelantha* and other genera included in this study are vulnerable or endangered due to illegal collecting. For this reason, no coordinates are included.

**Table S2** Pearson correlation coefficients calculated for the 19 bioclimatic variables. The interpretation of the keys is listed in table S4

**Table S3** Mean estimates for nine uncorrelated bioclimatic variables plus soil pH, calculated for each of the terminals that make up the calibrated tree.

**Table S4**. List of bioclimatic variables used in the analysis.

**Fig. S1.-** Character mapping of soil types and land forms along with their respective best fitting evolution model.

**TABLE S1**: Representative specimens of *Epithelantha* species and allied examined and corresponding GenBank accession numbers (*petL-psbE, psbA-trnH, trnL-F, trnQ-rps16*). *— missing sequence. The voucher was deposited in MEXU. *Epithelantha* and other genera included in this study are vulnerable or endangered due to illegal collecting. For this reason, no coordinates are included.

| **Specie** | **Collector** | **Location** | ***petL-psbE*** | ***psbA-trnH*** | ***trnL-F*** | ***trnQ-rps16*** |
| --- | --- | --- | --- | --- | --- | --- |
| *Ariocarpus trigonus* | S. Arias 1993 | MX. Tamaulipas | MK284027 | MK284089 | MK284149 | MK284208 |
| *Coryphantha calipensis* | B. Vázquez-Benítez 2555 | MX. Oaxaca | MK284029 | MK284091 | MK284151 | MK284210 |
| *Echinocactus platyacanthus* | S. Arias 1679 | MX. Querétaro | MK284037 | MK284099 | MK284159 | MK284218 |
| *Epithelantha bokei* | D. Aquino 318 | MX. Coahuila | MK283983 | MK284044 | MK284104 | MK284165 |
| *E. cryptica* | D. Aquino 355 | MX. Coahuila | MK283993 | MK284054 | MK284114 | MK284175 |
| *E. greggii* | D. Aquino 329 | MX. Nuevo León | MK284008 | MK284069 | MK284129 | MK284190 |
| *E. ilariae* | D. Aquino 323 | MX. Nuevo León | MK283987 | MK284048 | MK284108 | MK284169 |
| *E. micromeris* | D. Aquino 333 | MX. Nuevo Léon | MK283996 | MK284057 | MK284117 | MK284178 |
| *E. pachyrhiza* | D. Aquino 305 | MX. Coahuila | MK284013 | MK284074 | MK284134 | MK284195 |
| *E. polycephala* | D. Aquino 352 | MX. Coahuila | MK284019 | MK284080 | MK284140 | MK284201 |
| *E. potosina* | D. Aquino 383 | MX. San Luis Potosí | MK283990 | MK284051 | MK284111 | MK284172 |
| *E. pulchra* | D. Aquino 311 | MX. Nuevo León | MK284016 | MK284077 | MK284137 | MK284198 |
| *E. spinosior* | D. Aquino 334 | MX. Nuevo León | MK284001 | MK284062 | MK284122 | MK284183 |
| *Kadenicarpus horripilus* | J. M. Chalet 201 | MX. Hidalgo | MK284023 | MK284084 | MK284144 | MK284205 |
| *K. pseudomacrochele* | TCG 9101 | MX. Hidalgo | MK284022 | MK284083 | MK284143 | MK284204 |
| *Lophophora williamsii* | S. Arias 1849 | MX. San Luis Potosí | MK284026 | MK284088 | MK284148 | **—** |
| *Mammillaria mystax* | D. Aquino 225 | MX. Puebla | MK284033 | MK284095 | MK284155 | MK284214 |
| *Rapicactus beguinii* | S. Arias 1854 | MX. Nuevo León | MK284024 | MK284086 | MK284146 | MK284207 |
| *Strombocactus disciformis* | S. Arias 1738 | MX. Querétaro | MK284028 | MK284090 | MK284150 | MK284209 |
| *Turbinicarpus alonsoi* | A. García 24 | MX. Guanajuato | MK284021 | MK284082 | MK284142 | MK284203 |

**TABLE S2**. Pearson correlation coefficients calculated for the 19 bioclimatic variables. The interpretation of the keys is listed in table S4

|  | **bio1** | **bio2** | **bio3** | **bio4** | **bio5** | **bio6** | **bio7** | **bio8** | **bio9** | **bio10** | **bio11** | **bio12** | **bio13** | **bio14** | **bio15** | **bio16** | **bio17** | **bio18** | **bio19** |
| --- | --- | --- | --- | --- | --- | --- | --- | --- | --- | --- | --- | --- | --- | --- | --- | --- | --- | --- | --- |
| bio1 | 1 | 0.9901 | 0.991 | -0.100 | -0.049 | 0.1012 | -0.110 | -0.019 | 0.1199 | -0.039 | 0.100 | -0.011 | -0.067 | -0.014 | -0.17 | -0.029 | 0.046 | -0.023 | 0.0851 |
| bio2 | 0.990 | 1 | 0.996 | -0.115 | -0.106 | 0.0083 | -0.076 | -0.100 | 0.0873 | -0.134 | 0.032 | 0.0041 | -0.092 | -0.010 | -0.20 | -0.014 | 0.063 | 0.0170 | 0.1301 |
| bio3 | 0.991 | 0.9965 | 1 | -0.135 | -0.130 | 0.0446 | -0.119 | -0.112 | 0.0965 | -0.138 | 0.054 | 0.0136 | -0.070 | -0.004 | -0.18 | -0.005 | 0.075 | 0.0176 | 0.1361 |
| bio4 | -0.10 | -0.115 | -0.135 | 1 | 0.563 | -0.642 | 0.8647 | 0.619 | -0.507 | 0.704 | -0.72 | -0.607 | -0.343 | -0.453 | 0.100 | -0.524 | -0.617 | -0.554 | -0.534 |
| bio5 | -0.04 | -0.106 | -0.130 | 0.5633 | 1 | 0.0234 | 0.6377 | 0.884 | 0.2286 | 0.8771 | 0.070 | -0.210 | -0.054 | -0.196 | 0.191 | -0.167 | -0.285 | -0.247 | -0.303 |
| bio6 | 0.101 | 0.0083 | 0.044 | -0.642 | 0.023 | 1 | -0.755 | 0.054 | 0.6499 | 0.0552 | 0.950 | 0.3864 | 0.397 | 0.3048 | 0.087 | 0.337 | 0.363 | 0.2235 | 0.1756 |
| bio7 | -0.11 | -0.076 | -0.119 | 0.8647 | 0.637 | -0.755 | 1 | 0.537 | -0.350 | 0.5326 | -0.68 | -0.435 | -0.342 | -0.363 | 0.058 | -0.370 | -0.467 | -0.334 | -0.334 |
| bio8 | -0.01 | -0.100 | -0.112 | 0.6196 | 0.884 | 0.0548 | 0.5376 | 1 | 0.0837 | 0.9381 | 0.047 | -0.440 | -0.242 | -0.361 | 0.038 | -0.406 | -0.463 | -0.446 | -0.539 |
| bio9 | 0.119 | 0.0873 | 0.096 | -0.507 | 0.228 | 0.6499 | -0.350 | 0.083 | 1 | 0.0164 | 0.740 | 0.3401 | 0.247 | 0.1818 | 0.213 | 0.335 | 0.249 | 0.2464 | 0.2815 |
| bio10 | -0.03 | -0.134 | -0.138 | 0.7040 | 0.877 | 0.0552 | 0.5326 | 0.938 | 0.0164 | 1 | -0.02 | -0.416 | -0.123 | -0.310 | 0.167 | -0.365 | -0.453 | -0.480 | -0.523 |
| bio11 | 0.100 | 0.0326 | 0.054 | -0.723 | 0.070 | 0.9506 | -0.685 | 0.047 | 0.7404 | -0.020 | 1 | 0.4450 | 0.347 | 0.3246 | 0.015 | 0.378 | 0.419 | 0.3188 | 0.2374 |
| bio12 | -0.01 | 0.0041 | 0.013 | -0.607 | -0.210 | 0.3864 | -0.435 | -0.440 | 0.3401 | -0.416 | 0.445 | 1 | 0.879 | 0.8887 | 0.242 | 0.971 | 0.883 | 0.8774 | 0.7838 |
| bio13 | -0.06 | -0.092 | -0.070 | -0.343 | -0.054 | 0.3979 | -0.342 | -0.242 | 0.2475 | -0.123 | 0.347 | 0.8797 | 1 | 0.8072 | 0.526 | 0.906 | 0.707 | 0.7073 | 0.5919 |
| bio14 | -0.01 | -0.010 | -0.000 | -0.453 | -0.196 | 0.3048 | -0.363 | -0.361 | 0.1818 | -0.310 | 0.324 | 0.8887 | 0.807 | 1 | 0.066 | 0.818 | 0.910 | 0.7462 | 0.7621 |
| bio15 | -0.17 | -0.200 | -0.188 | 0.1001 | 0.191 | 0.0876 | 0.0580 | 0.038 | 0.2133 | 0.1674 | 0.015 | 0.2426 | 0.526 | 0.0662 | 1 | 0.422 | -0.11 | 0.1628 | -0.111 |
| bio16 | -0.02 | -0.014 | -0.005 | -0.524 | -0.167 | 0.3378 | -0.370 | -0.406 | 0.3354 | -0.365 | 0.378 | 0.9716 | 0.906 | 0.8181 | 0.422 | 1 | 0.771 | 0.8528 | 0.695 |
| bio17 | 0.046 | 0.0638 | 0.075 | -0.617 | -0.285 | 0.3633 | -0.467 | -0.463 | 0.2499 | -0.453 | 0.419 | 0.8833 | 0.707 | 0.9106 | -0.11 | 0.771 | 1 | 0.7947 | 0.9009 |
| bio18 | -0.02 | 0.017 | 0.017 | -0.554 | -0.247 | 0.2235 | -0.334 | -0.446 | 0.246 | -0.480 | 0.318 | 0.8774 | 0.707 | 0.7462 | 0.162 | 0.852 | 0.794 | 1 | 0.742 |
| bio19 | 0.085 | 0.1301 | 0.136 | -0.534 | -0.303 | 0.1756 | -0.334 | -0.539 | 0.2815 | -0.523 | 0.237 | 0.7838 | 0.591 | 0.7621 | -0.11 | 0.695 | 0.900 | 0.7422 | 1 |

**TABLE S3**. Mean estimates for nine uncorrelated bioclimatic variables plus soil pH, calculated for each of the terminals that make up the calibrated tree.

| **Taxon** | **bio1** | **bio3** | **bio4** | **bio9** | **bio11** | **bio12** | **pH** |
| --- | --- | --- | --- | --- | --- | --- | --- |
| *Epithelantha bokei* | 19.1875 | 52.3125 | 50.3006 | 14.5125 | 12.2687 | 311.6875 | 7.9483 |
| *E. cryptica* | 19.6000 | 49.6666 | 53.2000 | 13.8666 | 12.3333 | 309.3333 | 7.9356 |
| *E. greggii* | 20.6914 | 49.3142 | 52.5860 | 15.7457 | 13.4714 | 279.1714 | 7.9643 |
| *E. ilariae* | 22.0500 | 44.7500 | 56.6300 | 15.9500 | 14.2250 | 544.5000 | 7.7728 |
| *E. micromeris* | 17.1750 | 51.5625 | 55.7450 | 13.0437 | 9.7937 | 374.9375 | 7.9297 |
| *E. pachyrhiza* | 20.4333 | 51.0000 | 50.0866 | 15.0666 | 13.6000 | 302.0000 | 7.9668 |
| *E. polycephala* | 19.2500 | 51.0000 | 46.1750 | 14.3250 | 12.9750 | 254.5000 | 8.0324 |
| *E. potosina* | 19.3666 | 63.0000 | 30.4983 | 18.2666 | 14.8666 | 493.0000 | 7.8477 |
| *E. pulchra* | 20.7142 | 49.2857 | 48.3414 | 15.6714 | 13.9571 | 333.4285 | 7.9012 |
| *E. spinosior* | 19.3000 | 50.4285 | 45.1171 | 14.4000 | 13.1000 | 365.1428 | 7.9840 |
| *Turbinicarpus* | 17.9000 | 64.0000 | 29.2800 | 17.2000 | 17.2000 | 632.0000 | 7.0168 |
| *Kadenicarpus pseudomacrochele* | 18.1000 | 66.8000 | 23.9180 | 15.4000 | 14.8400 | 482.2000 | 7.1877 |
| *K. horripilus* | 21.3000 | 64.0000 | 24.8000 | 14.5000 | 14.5000 | 440.0000 | 7.0051 |
| *Ariocarpus* | 19.5274 | 58.6263 | 39.8185 | 15.5318 | 13.9714 | 410.2967 | 7.8128 |
| *Strombocactus* | 21.2818 | 63.2727 | 28.9818 | 18.1272 | 17.2363 | 586.8181 | 7.0779 |
| *Rapicactus* | 17.4846 | 61.6923 | 34.5838 | 13.7884 | 12.6692 | 471.0000 | 7.8486 |
| *Lophophora* | 19.6732 | 61.5070 | 35.7250 | 16.2901 | 14.6845 | 407.1408 | 7.6118 |
| *Mammillaria* | 19.0948 | 64.9124 | 25.3548 | 16.6916 | 15.5761 | 613.9008 | 6.9824 |
| *Coryphantha* | 18.6626 | 63.3123 | 29.1519 | 15.7207 | 14.5965 | 516.7085 | 7.2571 |
| *Echinocactus* | 18.9000 | 63.7400 | 31.8000 | 16.0000 | 14.4000 | 453.1800 | 7.6100 |

**TABLE S4**. List of bioclimatic variables used in the analysis.

| Key | Description of variables |
| --- | --- |
| Bio1 | Annual mean temperature |
| Bio2 | Mean diurnal range (mean of monthly (max temp - min temp)) |
| Bio3 | Isothermality (BIO2/BIO7) (* 100) |
| Bio4 | Temperature Seasonality (standard deviation *100) |
| Bio5 | Max temperature of warmest month |
| Bio6 | Min temperature of coldest month |
| Bio7 | Temperature annual range (BIO5-BIO6) |
| Bio8 | Mean temperature of wettest quarter |
| Bio9 | Mean temperature of driest quarter |
| Bio10 | Mean temperature of warmest quarter |
| Bio11 | Mean temperature of coldest quarter |
| Bio12 | Annual precipitation |
| Bio13 | Precipitation of wettest month |
| Bio14 | Precipitation of driest month |
| Bio15 | Precipitation seasonality (Coefficient of variation) |
| Bio16 | Precipitation of wettest quarter |
| Bio17 | Precipitation of driest quarter |
| Bio18 | Precipitation of warmest quarter |
| Bio19 | Precipitation of coldest quarter |


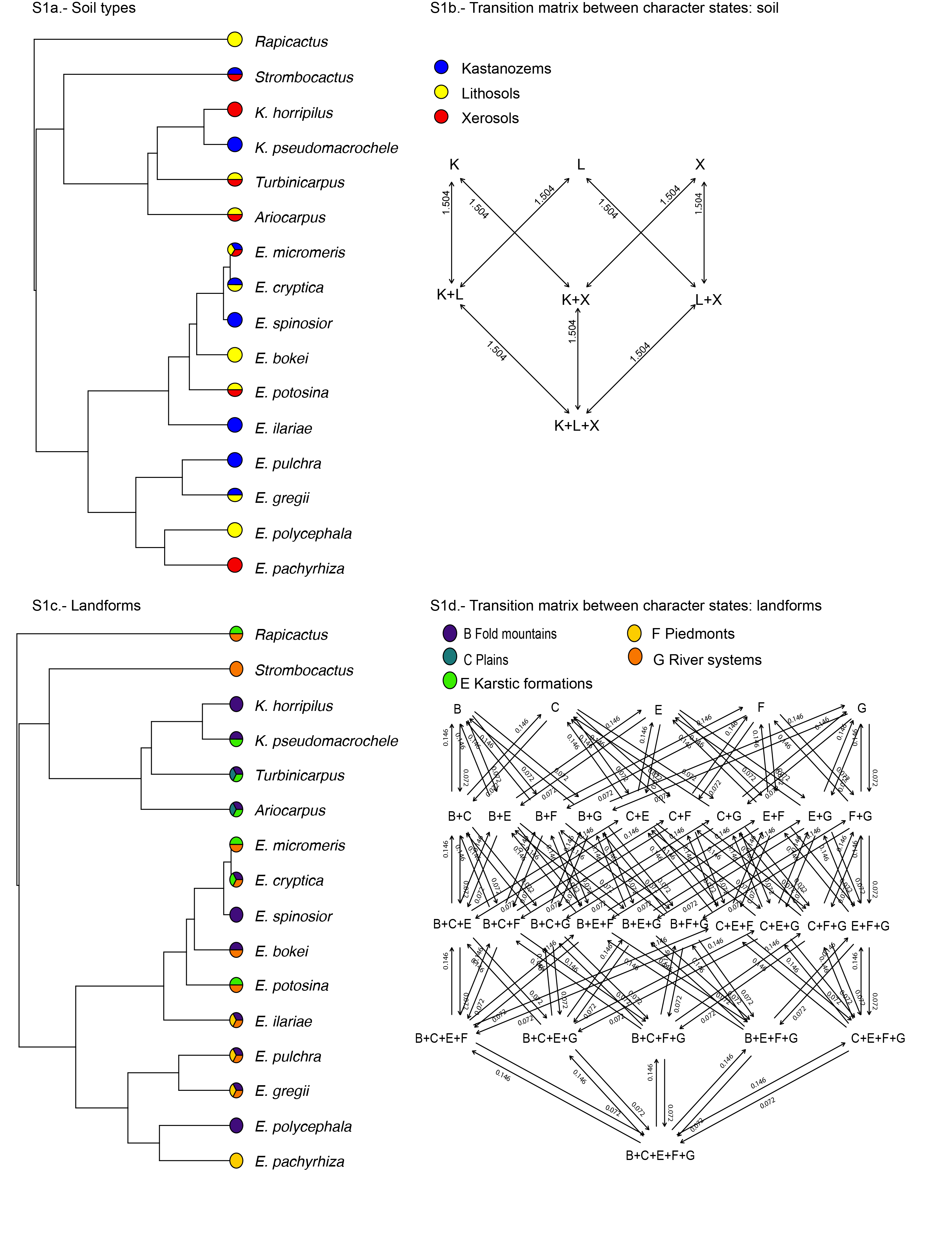
**Fig. S1.-** Character mapping of soil types and land forms along with their respective best fitting evolution model.
